# A cost-effective system for differentiation of intestinal epithelium from human induced pluripotent stem cells

**DOI:** 10.1038/srep17297

**Published:** 2015-11-30

**Authors:** Soichiro Ogaki, Mayu Morooka, Kaito Otera, Shoen Kume

**Affiliations:** 1 Stem Cell Biology, Institute of Molecular Embryology and Genetics, Kumamoto University, Honjo 2-2-1, Kumamoto 860-0811, Japan; 2 Research Fellow of Japan Society for the Promotion of Science; 3 Department of Biological Information, Graduate School of Bioscience and Biotechnology, Tokyo Institute of Technology, 4259-B-25 Nagatsuta-cho, Midori-ku, Yokohama-shi, Kanagawa, 226-8503, Japan.; 4 Japan Agency for Medical Research and Development, Department of Clinical Research and Trials, Office of Regulatory Science and Clinical Research Support; 5 Division of Pharmacology, National Institute of Health Sciences.

## Abstract

The human intestinal epithelium is a useful model for pharmacological studies of absorption, metabolism, drug interactions, and toxicology, as well as for studies of developmental biology. We established a rapid and cost effective system for differentiation of human induced pluripotent stem (iPS) cells into definitive endoderm (DE) cells. In the presence of dimethyl sulfoxide (DMSO), a low concentration of Activin at 6.25 ng/ml is sufficient to give a similar differentiation efficiency with that using Activin at 100 ng/ml at the presence of Wnt activator. In the presence of DMSO, Activin at low concentration triggered hiPS cells to undergo differentiation through G1 arrest, reduce apoptosis, and potentiate activation of downstream targets, such as SMAD2 phosphorylation and SOX17 expression. This increased differentiation into CDX2 + SOX17 + DE cells. The present differentiation procedure therefore permits rapid and efficient derivation of DE cells, capable of differentiating into intestinal epithelium upon BIO and DAPT treatment and of giving rise to functional cells, such as enterocytes.

The intestinal epithelium is derived from the definitive endoderm (DE), which then gives rise to functional types of secretory cells, such as goblet cells, enteroendocrine cells, and paneth cells, or to absorptive cells known as enterocytes, which play important roles in nutrient absorption and drug metabolism^1^,^2^,^3^.

During mouse embryonic development, regionalization occurs immediately after gastrulation. *Sox17* and *Cdx2* encode key transcription factors for establishing the regional specific identity of the intestinal epithelium. Sox17-expressing cells represent posterior endoderm that start to express Cdx2 at embryonic day 8.5 (E8.5)^4^,^5^. *Sox17* mutant mice exhibit abnormal midgut and hindgut formation^6^. Therefore, Sox17 and Cdx2 are important molecules that mark posterior DE formation. DE specific-*Cdx2* mutant mice show conversion of the intestinal epithelium into an esophageal fate^7^. At E10.5, the immature gut epithelium is characterized by villin expression in the cytoplasm^8^. Around E14.5, the epithelia then differentiate into immature enterocytes that exhibit alkali phosphatase (ALP) activity^7^.

For drug development, the human colon cancer cell line Caco-2 is widely used as a model of the intestinal epithelium for testing absorptive and metabolic functions. However, Caco-2 cells show low enzymatic activity, and show cell line-to-cell line differences in their properties^9^,^10^. Therefore, there is a need to establish novel *in vitro* models as substitutes for Caco-2 cells in drug tests. Recently, using Matrigel supplemented with various growth factors and chemical inhibitors, 3-dimensional systems for organ culture using intestinal stem cells (ISCs) have been reported^11^. Organoid culture systems for intestinal differentiation from human induced pluripotent stem (hiPS) cells have also been reported^12^,^13^,^14^. However, 3-dimensional organoids form with their apical surfaces residing in the inner domain and their basement membranes in the outer layer, and are surrounded by an extracellular matrix. Using organoids for pharmacological and toxicological studies will require injection of substrates into individual organoids for exposure to the apical membrane. Therefore, there is a need to establish a monolayer system to differentiate hiPS cells into the intestine. In previous reports, 100 ng/ml Activin was used to differentiate hiPS cells into DE, then high concentrations of Wnt and FGF were used to induce posterior DE. For differentiating intestinal cells in a cost effective manner, we previously established a 2-dimensional procedure for intestinal epithelial differentiation from mouse and human embryonic stem (ES) cells using low molecule compounds. After DE differentiation, addition of 6-bromoindirubin-3′-oxime (BIO), a glycogen synthase kinase (GSK)-3β inhibitor, and DAPT, a γ-secretase inhibitor, synergistically induced CDX2-expressing posterior definitive endodermal cells, which then differentiated into four mature intestinal cell types, namely enterocytes, goblet cells, enteroendocrine cells, and paneth cells^15^,^16^.

Chemical compounds that increase DE differentiation from pluripotent stem cells have been described^17^,^18^,^19^. Many of chemical compounds are typically dissolved in DMSO, which is used as a solvent. However, DMSO itself exerts effects on cells^20^,^21^,^22^,^23^,^24^,^25^. DMSO has been used for treatment of diseases, such as amyloidosis, for its anti-inflammatory and reactive oxygen species scavenger activities^24^, and has been shown to promote leukemic cell differentiation^26^. DMSO has also been used to promote differentiation. Pre-treatment with DMSO before differentiation was shown to promote ectoderm, mesoderm and DE differentiation of hiPS cells. Under these protocols, 100 ng/ml Activin was used for DE differentiation^12^,^15^,^18^,^19^,^27^,^28^,^29^,^30^,^31^. Here, we found that DMSO decreased the threshold for Activin, so that 6.25 ng/ml Activin was sufficient for the induction of DE differentiation at a high efficiency. We examined the underlying molecular mechanism. Wnt activators previously reported as the promoter of DE differentiation was not able to substitute DMSO. Our cost-effective protocol could be adapted for differentiating into not only intestinal but also hepatic, pancreatic and anterior foregut lineages.

## Results

### DMSO promotes Activin-induced definitive endodermal differentiation from hiPS cells

**Figure 1:**
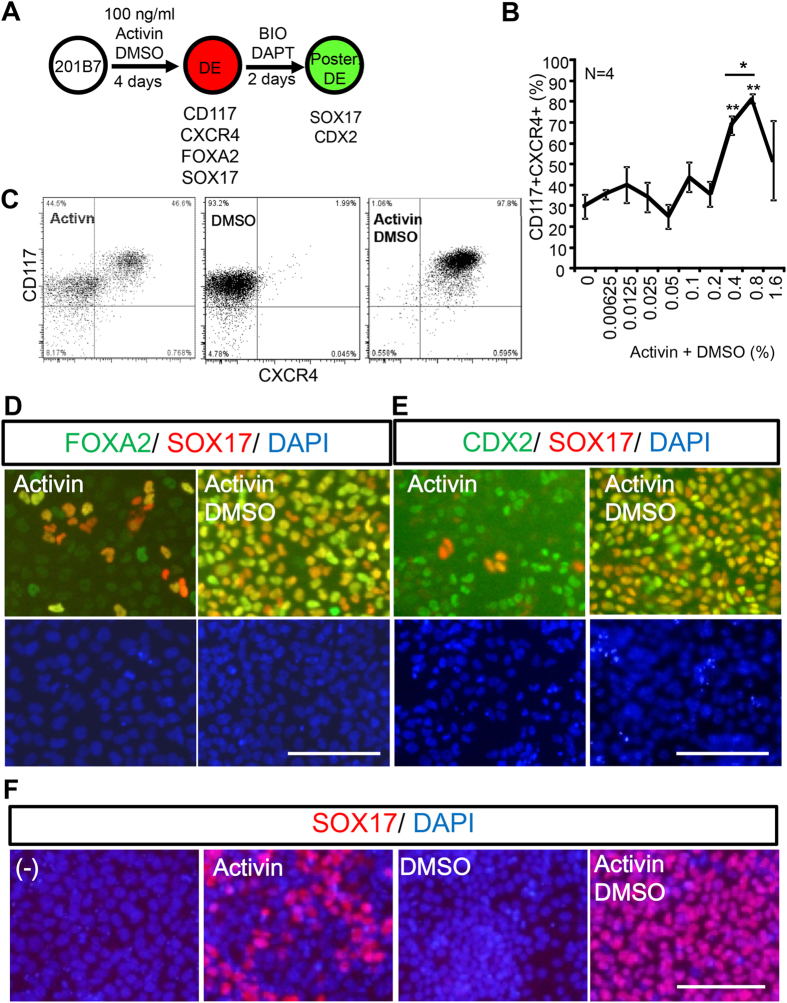
Dimethyl sulfoxide (DMSO) promoted endodermal differentiation from 201B7 hiPSCs. Human 201B7 iPS cells were differentiated into definitive endoderm (DE) at a graded concentration of DMSO, and the resultant DE was characterized. (**A**) Scheme for hiPS cell differentiation. 201B7, hiPS cells, were differentiated into DE cells with Activin and DMSO. Then, BIO and DAPT were added to induce posterior definitive endoderm (Post. DE). (**B,C**) Flow cytometry analysis. Graded concentrations of DMSO were tested for DE differentiation, and 0.8% DMSO had the strongest effect for potentiating differentiation into CD117+CXCR4+ cells (**B**). A representative result at 0.8% DMSO is shown (**C**). (**D–F**) Immunocytochemical analysis. FOXA2 (green), SOX17 (red), CDX2 (green), DAPI (blue). (**D,E**) DMSO treatment potentiated DE differentiation, and increased the proportion of FOXA2+SOX17+ endodermal cells (**D**) and CDX2+SOX17+ posterior endoderm (**E**). (**F**) Activin was required for DE (indicated by SOX17+ staining) differentiation. DMSO alone could not induce DE differentiation from hiPS cells. Scale bar; 50 μm.

Previously, in an attempt to establish a screening procedure for chemicals that potentiate DE differentiation, we found that the widely used solvent DMSO itself produced potentiation effects. In this study, we used a hiPS cell line, 201B7, to evaluate DMSO-mediated potentiation of DE differentiation. The experimental scheme is shown in Fig. 1A. 201B7 cells were triggered to adopt DE differentiation in media containing Activin (100 ng/ml) for 4 days, in the presence of graded concentrations of DMSO (0–1.6%), then exposed simultaneously to 6-bromoindirubin-3′-oxime (BIO), a glycogen synthase kinase (GSK)-3β inhibitor, and DAPT, a γ-secretase inhibitor, gave rise to CDX2-expressing posterior DE cells.

After the initial 4-day culture with Activin and DMSO, the cells were assayed for DE differentiation by flow cytometry. In contrast to the control condition (without DMSO), in which the proportion of CD117+ CXCR4+ endodermal cells was 29.6 ± 5.65%, the presence of 0.8% DMSO yielded the greatest DE differentiation efficiency for 100 ng/ml of Activin to induce up to 81.3 ± 2.17% CD117 + CXCR4 + cells (Fig. 1B,C). Without Activin, 0.8% DMSO could not induce CD117 + CXCR + DE cells from 201B7 (Fig. 1B). DMSO-mediated potentiation of DE differentiation by Activin (100 ng/ml) was conserved in another hiPS cell line, Toe, although 6 days were required for DE differentiation of Toe cells (Supplementary Fig. S1).

We then examined whether DE cells further differentiated into posterior DE cells. After 2 days of incubation with BIO and DAPT, SOX17 + gave rise. These cells were FOXA2 + SOX17 + (Fig. 1D, right panels) or CDX2 + SOX17 + Fig. 1E, right panels), thus revealing that the induced DE cells are of a posterior DE nature. However, when initial differentiation were performed only with Activin and in the absence of DMSO, many FOXA2-SOX17- and CDX2 + SOX17- cells were observed (Fig. 1D,E, left panels). Since CDX2 is expressed in both DE and non-DE cells, CDX2 + SOX17- cells appear to represent non-DE cells. DMSO alone did not induce DE differentiation (Fig. 1F). Collectively, our results indicate that DMSO enhanced Activin-mediated DE differentiation of human iPS cells.

### DMSO enables a low concentration of Activin to induce definitive endodermal cells from hiPS cells

**Figure 2:**
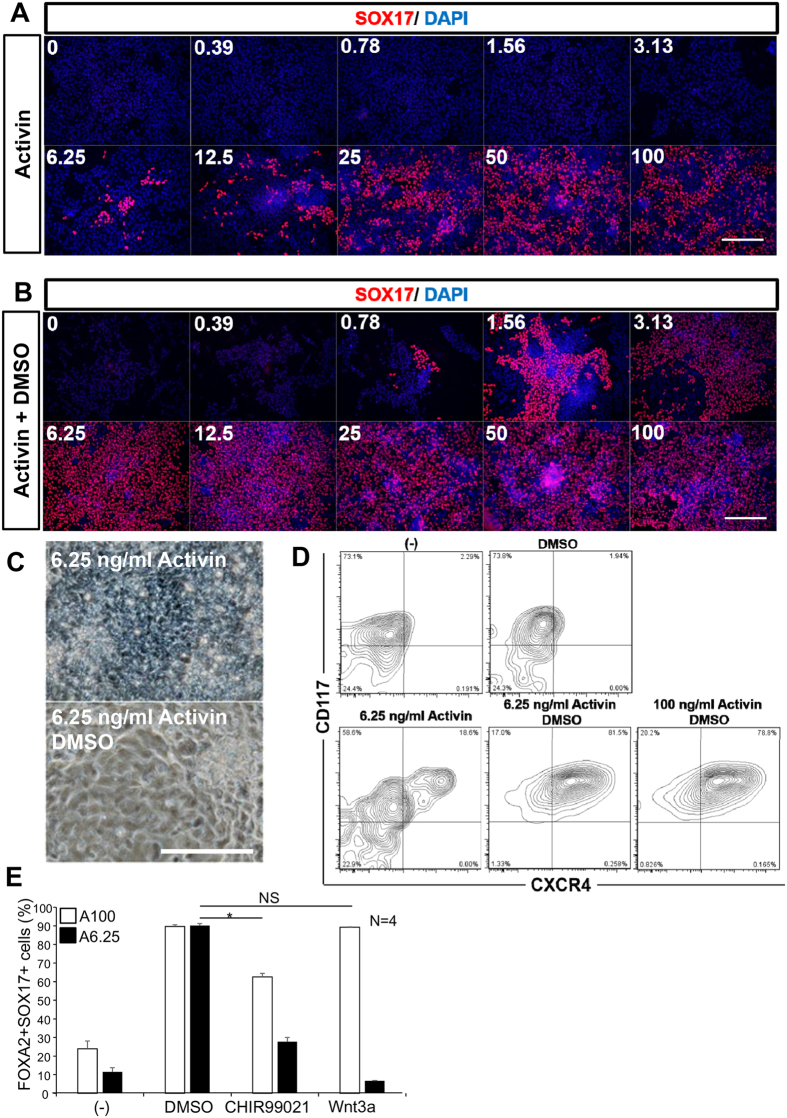
DMSO allowed a low concentration of Activin to trigger 201B7 cells to differentiate into DE. The concentration of Activin required to induce DE differentiation from 201B7 iPS cells in the presence of DMSO was examined. (**A,B**) In the presence of DMSO, Activin can induce DE differentiation at much lower concentrations, as indicated by (**A,B**) immunocytochemical analysis of SOX17 (red) staining, (**C**) cell morphology, (**D**) flow cytometry analysis of CD117 and CXCR4. (**E**) DMSO is more effective than CHIR99021 or Wnt3a to promote Activin (at 100 or 6.25 ng/ml) mediated DE differentiation. FOXA2+SOX17+ cells were quantified and compared. A100, Activin at 100 ng/ml; A6.25, Activin at 6.25 ng/ml. Scale bar; (**A,B**) 100 μm, (**C**) 20 μm.

We then examined whether DMSO enabled a low concentration of Activin to induce DE cells from hiPS cells. Activin was used at 100 ng/ml for DE differentiation of human pluripotent stem cells^12^,^15^,^19^,^29^,^31^,^32^. We performed differentiation with graded concentrations of Activin (0 ng/ml–100 ng/ml) in the presence or absence of 0.8% DMSO. After triggering DE differentiation of hiPS cells with DE differentiation medium, the number of SOX17+ cells increased in an Activin concentration-dependent manner (Fig. 2A). Without DMSO, very few SOX17+ cells appeared at low Activin concentrations (Fig. 2A). In contrast, in the presence of 0.8% DMSO, SOX17+ cells appeared at low concentrations of Activin (from 0.78 ng/ml) and reached a plateau at 6.25 ng/ml of Activin (Fig. 2B). The induced DE, showing endodermal morphology, was positive for both CD117 and CXCR4 (Fig. 2C,D). Potentiation of DE differentiation by DMSO at low concentration of Activin was also observed with Toe hiPS cells (Supplementary Fig. S2). Next, we divided the 96-h differentiation period into two time windows (the first and second halves to examine the time period of DMSO exposure required (Supplementary Fig. S3). The highest proportion of SOX17+ cells was observed when DMSO was added throughout the 96 h of differentiation. Therefore, continuous exposure to DMSO was required to potentiate DE differentiation (Supplementary Fig. S3).

We then compared the potentiation effect of DMSO with the reported DE promoter, CHIR99021 and Wnt3a. Activin at 6.25 ng/ml with DMSO is sufficient to induce DE differentiation at a similar extent triggered by Activin at 100 ng/ml with Wnt3a. However, Activin at 6.25 ng/ml added with CHIR99021 or Wnt3a at the absence of DMSO were much less potent to induce DE (Fig. 2E). These results suggested that DMSO is more effective compared to Wnt3a or CHIR99021 to potentiate DE differentiation by Activin at 6.25 ng/ml. Moreover, we found that DMSO also potentiated mesodermal differentiation at low concentrations of both BMP and Activin from hiPS cells. At the presence of DMSO, mesoderm was induced at BMP 2.5 ng/ml and Activin 6.25 ng/ml, assayed by the expression of BRACHYURY and MESP1 (Supplementary Fig. S4). These results suggested that the protocol utilizing DMSO enables a cost effective not only for DE but also for mesoderm differentiation from hiPS cells.

### DMSO promoted definitive endodermal differentiation by reducing the Activin threshold and by decreasing proliferation and apoptosis

**Figure 3:**
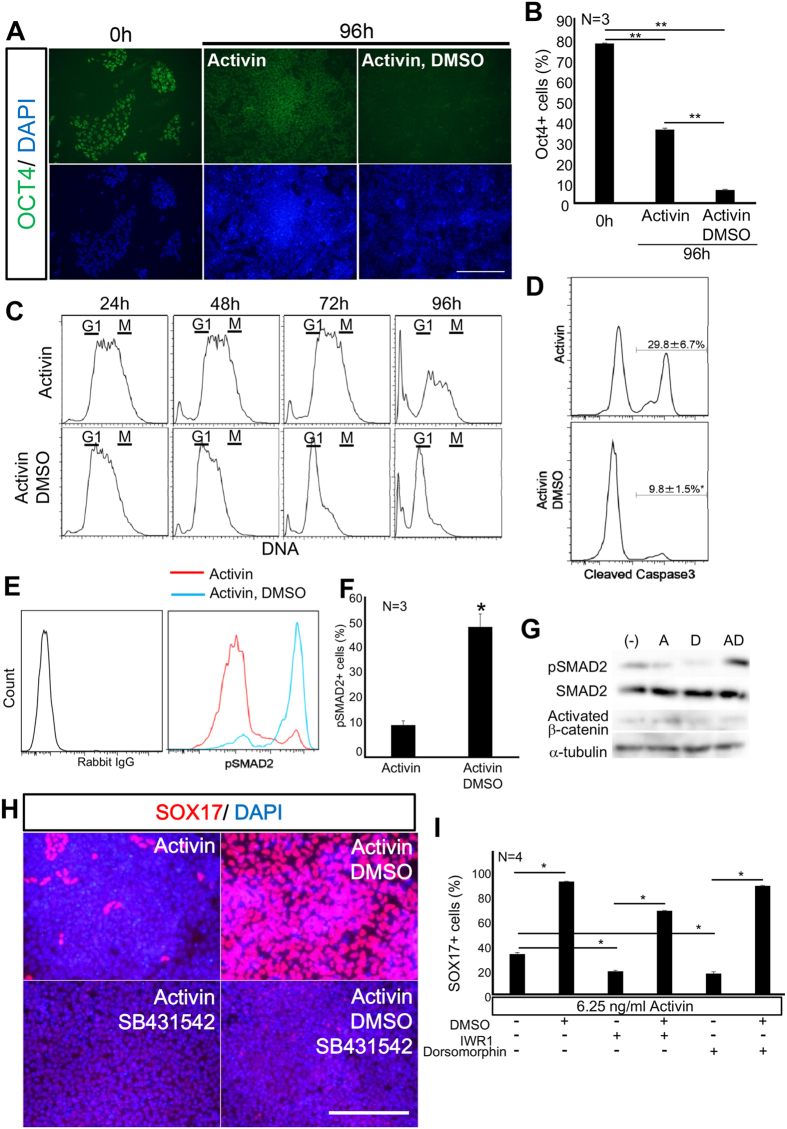
DMSO promoted a low dose Activin-mediated decrease in Oct4 expression and apoptosis, and increased the G1 population and SMAD2 phosphorylation. The molecular mechanism underlying DMSO potentiation of low dose (6.25 ng/ml) Activin-induced DE differentiation was analysed. (**A,B**) DMSO decreased the proportion of OCT4-expressing cells induced by Activin at low dose, as analysed by immunocytochemistry. (**C**) DMSO arrested the cell cycle and decreased cell death, as revealed by flow cytometry to measure DNA quantities using DyeCycle. (**D**) Flow cytometric analysis of cleaved caspase3 revealed that DMSO inhibited apoptosis, triggered by a low dose of Activin. (**E,F**) Flow cytometric analysis of phosphorylated SMAD2. DMSO promoted SMAD2 phosphorylation, induced by a low dose Activin. (**G**) Western blot analysis. DMSO increased pSMAD2 in cells induced by Activin at a low dose. However, Activated β-catenin was unchanged. (**H**) Suppression of Activin signalling by SB231542 completely inhibited DE differentiation by DMSO, as shown by immunocytochemistry. (**I**) Wnt and BMP signalling were not required for DE differentiation by DMSO, as revealed by immuncytochemical analysis of SOX17. Scale bar; 50 μm.

To understand the mechanism underlying the action of DMSO, OCT3/4 expression was examined at 0 h and 96 h after differentiation was triggered by Activin. At 96 h, DMSO treatment further decreased the proportion of OCT4-expressing cells (Fig. 3A,B), as determined by immunocytochemical analysis. Previously, DMSO was reported to induce cell cycle arrest^31^. We analysed the cell cycle stage of differentiating cells using DyeCycle for DNA content analysis in living cells. Cells differentiated by Activin in the presence of DMSO showed promotion of G1 arrest (Fig. 3C) and a decrease in the proportion of cleaved caspase3+ cells, indicating a decrease in apoptosis (Fig. 3D).

Activin signalling is mediated through activation of its receptor and phosphorylation of SMAD2^33^. We measured the proportion of cells with phosphorylated SMAD2 by flow cytometry^34^. At day 5, the proportion of Activin-triggered phosphorylated SMAD2 was significantly increased by DMSO treatment, from 12% to 50% (Fig. 3E–G). Suppression of Activin signalling by SB431542 completely inhibited DE differentiation, as shown by the decrease in SOX17+ cells (from 93% to less than 0.01%), even in the presence of DMSO (Fig. 3H).

Wnt and BMP signalling are also reported to mediate DE differentiation^19^. We examined whether DMSO acted through modifying Wnt or BMP signalling, which are likely to be endogenously derived from differentiating ES cells. We examined the activation of WNT signalling. However, DMSO had no effect on activated β-catenin (Fig. 3G). Then, using IWR-1, a Wnt signalling inhibitor, or dorsomorphin, a BMP signalling inhibitor, DE differentiation was partially suppressed (Fig. 3I). However, even in the presence of dorsomorphin or IWR-1, DMSO promoted DE differentiation (Fig. 3I). Collectively, these results suggested that DMSO might potentiate DE differentiation through Activin signalling but not through Wnt or BMP signalling. DMSO could potentiate Activin signalling by lowering the threshold for activation of its receptors and downstream targets, such as SMAD2 phosphorylation and transcriptional activation of *SOX17*. The data indicate that DMSO may enable a low concentration of Activin to trigger differentiation by decreasing OCT4 expression, slowing proliferation of hiPS cells, and protecting cells from apoptosis.

### In the presence of DMSO, a low concentration of Activin rapidly directed differentiation into the intestinal epithelial lineage

**Figure 4:**
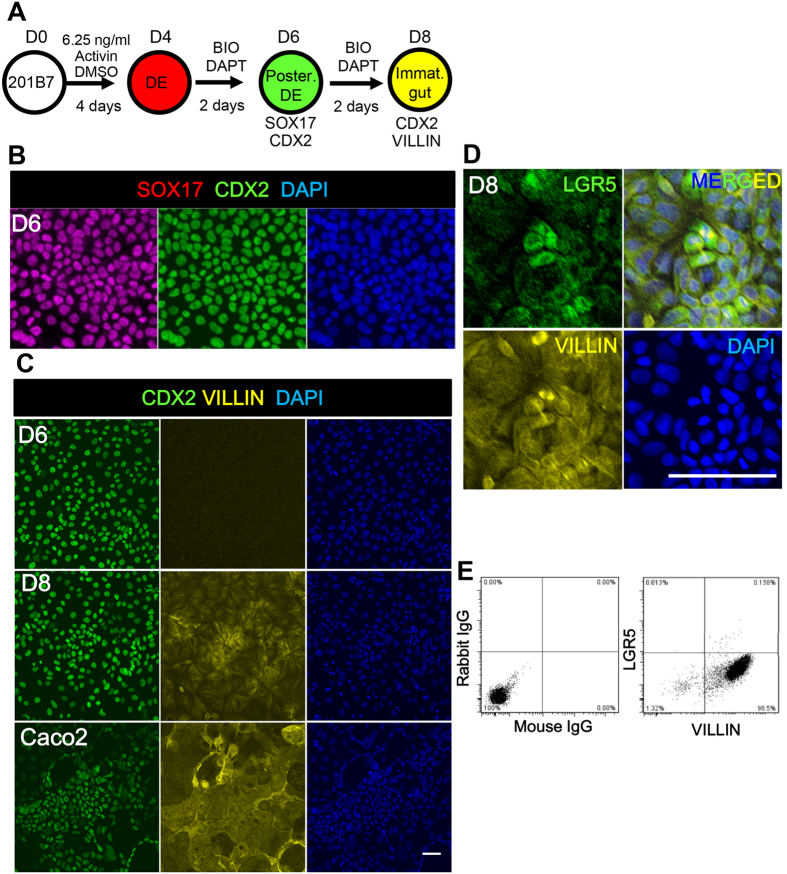
DE induced by 6.25 ng/ml Activin and DMSO further differentiated into immature gut epithelium. The ability of DE to differentiate into immature gut epithelium, triggered by a low dose of Activin and DMSO, was characterized. (**A**) The scheme for hiPS cell differentiation. After DE differentiation with 6.25 ng/ml Activin and DMSO, further incubation with BIO and DAPT induced differentiation into immature gut epithelium. (**B–E**) BIO and DAPT induced intestinal epithelium. (**B**) At day 6, SOX17+CDX2+ cells appeared. (**C–E**) At day 8, CDX2+ cells began to express VILLIN (**C**) and LGR5 (**D,E**), as shown by immunocytochemistry (**C,D**), and flow cytometry (**E**). Caco2 cells are used as a positive control for VILLIN expression. Scale bar; 50 μm.

To test the differentiation potency of the DE yielded by the present protocol, the DE was further differentiated into the intestinal epithelial lineage by addition of BIO and DAPT and cultured for another 2 to 4 days (Fig. 4A). At day 6, CDX2+SOX17+ posterior DE cells were observed (Fig. 4B, Supplementary Fig. S5). In mouse embryo, Villin is reportedly expressed in the cytoplasm in the immature gut epithelium at E10.5^8^, prior to its later apical expression restricted in the enterocytes. Here, we used VILLIN as a marker for immature gut epithelium. The CDX2+SOX17+ posterior endoderm cells further gave rise to CDX2+VILLIN+ immature gut epithelial cells on day 8 (Fig. 4C, D8), although the expression level was lower than that of the Caco2 cells, a human intestinal cell line showing comparable VILLIN expression to that of the human intestine^35^. Villin expression in Caco2 cells was also observed in the cytoplasm with fibrous staining, as previously reported^36^,^37^. Among these VILLIN+ cells, cells positive for LGR5, an intestinal stem cell marker, were observed (Fig. 4D,E). We also confirmed that the DE yielded by this protocol further differentiated into albumin- and CK19-expressing hepatoblasts^32^, PDX1-expressing pancreatic progenitor^38^ and SOX2-expressing anterior foregut cells^39^ (Supplementary Fig. S6), by following the reported protocol^40^,^41^. These data suggested that this protocol is useful for derivation not only of posterior but also of anterior endodermal lineages.

**Figure 5:**
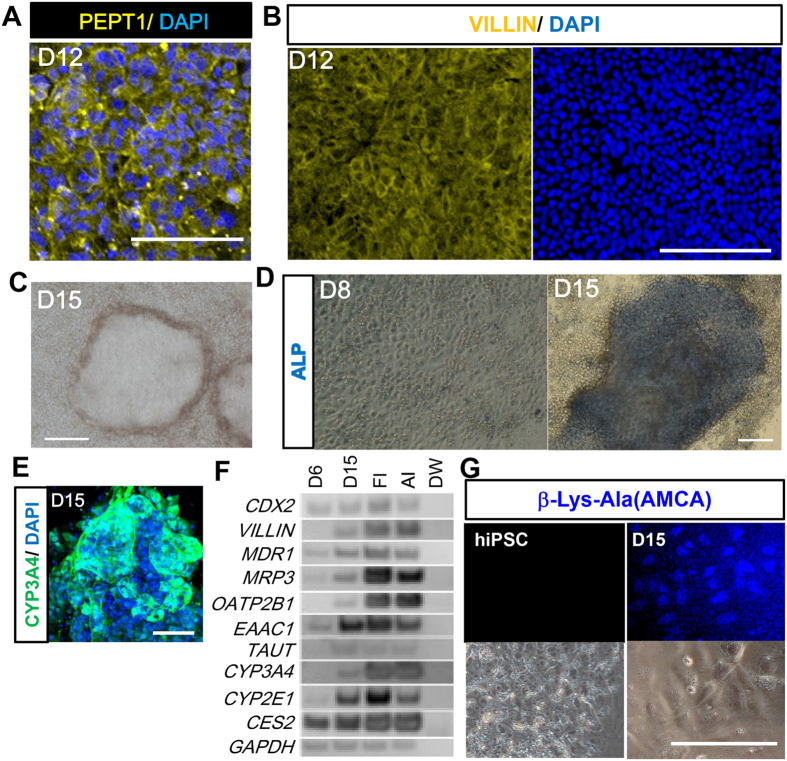
DE induced by 6.25 ng/ml of Activin and DMSO further differentiated into enterocytes. The ability of DE obtained by treatment with a low dose of Activin and DMSO to differentiate into enterocytes was characterized. (**A**) hiPS cell-derived cells expressed the intestinal transporter, PEPT1, at day 12. (**B**) Intestinal epithelium formed a dome-like structure, which resembles enterocyte morphology, at day 15. (**C–E**) These cells showed alkali phosphatase activity (ALP) (**C**) and expressed enterocyte markers, by immunocytochemistry (**D**) or RT-PCR (**E**). (**G**) β-Lys-Ala (AMCA) intake assayed at day 15. For primer sequences used, see Supplementary Table S1. The gels were cropped; for full gel images, see Supplementary Fig. 6. Scale bar; (**A**) 50 μm, (**B–D**) 100 μm. FI; Fetal Intestine. AI; Adult Intestine.

Next, we examined whether immature gut epithelial cells could differentiate into absorptive enterocytes. At day 12, the differentiated cells expressed an enterocyte-specific transporter, PEPT1 (Fig. 5A). At day 15, cells exhibited a dome-like structure, which is a characteristic feature of enterocytes (Fig. 5B), began to express enterocyte markers such as *MDR1*, *MRP3*, *OATP2B1*, *EAAC1*, *TAUT*, *CYP3A4*. *CYP2E1*, and *CES2*^42^ (Fig. 5C–E, Supplementary Fig. S6), and exhibited alkali phosphatase (ALP) activity (Fig. 5C). ALP activity is known as a functional assay for enterocytes^43^. We also confirmed that hiPS cells-derived enterocytes showed PEPT1 activities by assaying the intake of β-Ala-Lys (AMCA), a fluorescent dipeptides^44^ (Fig. 5G). These results therefore indicated that the DE cells induced using a low concentration Activin were potent to differentiate into enterocyte-like cells.

## Discussion

There is a need for intestinal epithelial cells derived from human iPS cells as model human cells for pharmacological and toxicological studies for drug development. Establishment of a low-cost differentiation protocol would be beneficial for conducting these studies. The intestinal epithelium is derived from the DE. Most of the reported DE differentiation protocols from human iPS cells use recombinant Activin at 100 ng/ml to induce differentiation into DE lineages^12^,^15^,^18^,^19^,^27^,^28^,^29^,^30^,^31^. Teo *et al*. concluded that Activin at 100 ng/ml combined with 3βM CHIR99021, a Gsk3β inhibitor, was a cost-effective protocol for DE differentiation from human iPS cells^19^. Thus, the protocol described here, which uses an even lower concentration of Activin (6.25 ng/ml), is the most cost effective protocol developed to date.

Activin is a member of the transforming growth factor beta (TGFβ) family. Activin binds to and activates the receptors ALK4 and ActR-II, which in turn phosphorylate Smad2. Phosphorylated Smad2 binds to Smad4, a co-activator, to regulate expression of target genes^33^. We found that in human iPS cells, DMSO permits a low dose of Activin (6.25 ng/ml) to phosphorylate SMAD2 and can induce SOX17+ cells indicative of DE differentiation, which can then be directed into the intestinal epithelial fate at high efficiency.

DMSO is frequently used as a solvent. DMSO is reported to regulate the cell cycle, differentiation, and apoptosis and to activate various signalling pathways. Treatment of human pluripotent stem (PS) cells with 1–2% DMSO prior to differentiation promotes differentiation as a result of G1 arrest^31^. Cell cycle progression is reportedly associated with the differentiation state of stem cells, and undifferentiated human PS cells arrested in early G1 exhibit a high propensity to trigger differentiation into mesoderm and DE, through the release of CDK4/6-mediated inhibition of Smad2 phosphorylation^45^. However, they did not examine the possibility to lower the thresholds of the growth factors for differentiation. A low concentration of Activin (10 ng/ml) with 0.75 μM PD0332991, a CDK4/6 inhibitor, induced DE differentiation of H9 human ES cells^45^. Here, we found that DMSO promoted G1 arrest and phosphorylation of Smad2. However, unlike the above-mentioned reports, in which cells were pre-treated with DMSO before differentiation, we showed that DMSO was required during all periods of differentiation and that PD0332991 could not substitute for DMSO to potentiate differentiation (S.O. unpublished data). Therefore, an unknown mechanism, other than G1 arrest, may be involved. These results show that DMSO potentiates a low concentration of Activin to induce iPS cells to adopt the DE fate, causes G1 arrest, and lowers the threshold for SMAD2 activation and subsequent *SOX17* transcriptional activation. In the presence of DMSO, Activin induced DE cells at a high efficiency, which were then capable of differentiating into the intestinal lineage following BIO and DAPT treatment.

The differentiated cells exhibited ALP activity and expressed drug transporters and metabolic enzymes, although at lower levels than are observed in cells of the adult human intestine. This protocol could be applied to other low-cost differentiation protocol for other endodermal lineages.

Our previous protocol for generating hiPS-derived intestinal cells required 20–30 days, using feeder cells^15^; here we have established a novel protocol to achieve rapid and efficient differentiation into intestinal epithelial cells that are capable of further differentiating into functional cells, such as enterocytes.

## Methods

### Cell Lines

We thank Dr. S. Yamanaka (Kyoto University, Kyoto, Japan) for providing the human iPS cell (hiPSC) line, 201B7. We thank Drs. M. Toyoda, N. Kiyokawa, H. Okita, Y. Miyagawa, H. Akutsu, and A. Umezawa (National Institute for Child Health and Development, Tokyo^46^) for providing the Toe hiPS cell line. Undifferentiated hiPS cells were maintained on a feeder layer of mouse embryonic fibroblasts (MEFs) in Knockout DMEM/F12 (Invitrogen) supplemented with 5 ng/ml FGF2 (Peprotech), 20% Knockout Serum Replacement (KSR, Invitrogen), 2 mM L-glutamine (L-Gln, Nacalai Tesque), 100 mM non-essential amino acids (NEAA, Invitrogen), 50 units/mL penicillin, 50 mg/ml streptomycin (PS, Nacalai Tesque), and 100 μM 2-mercaptoethanol (2-ME, Sigma-Aldrich) in 5% CO_2_. To passage hiPSCs, colonies were detached from the feeder layer by treating with 0.25% trypsin (Invitrogen) and 0.1 mg/ml collagenase IV (Gibco) in phosphate buffered saline (PBS) containing 20% KSR and 1 mM CaCl_2_ at 37 °C for 6 min, followed by adding culture medium and disaggregating ES cell clumps into smaller pieces (5–20 cells) by gently pipetting several times. HepG2 and Caco2 cells were maintained in DMEM supplemented with 10% FBS (Hyclone) and PS.

### Intestinal, hepatic, pancreatic and anterior foregut differentiation of hiPS cells

For differentiation studies, hiPS cells were pre-treated with 10 μM Y27632 (Wako) for 24 h, plated at 70,000 cells per well in 96-well plates or 210,000 cells per well in 24-well plates previously coated with BD Matrigel (BD Biosciences). hiPSCs were dissociated with 0.25% trypsin-EDTA and cultured in 4500 mg/L glucose DMEM (Invitrogen) supplemented with 10% fetal bovine serum (FBS, Hyclone), 2 mM L-Gln, 100 mM NEAA, PS, and 100 μM 2-ME for 1 day. Twenty-four hours after plating, cells were washed with PBS and the medium was changed to endodermal differentiation medium [4500 mg/L glucose DMEM (Invitrogen) supplemented with 0 ng/ml–100 ng/ml Activin A (R&D Systems), 2% B27 (Invitrogen), 0–1.6% dimethyl sulfoxide (DMSO) Hybri-max (Sigma), NEAA, L-Gln, PS, and 2-ME] for 4-6 days, then switched to either intestinal differentiation medium [DMEM supplemented with 5 μM BIO (Calbiochem), 10 μM DAPT (Peptide), 10% KSR, glucose 2000 mg/ml, NEAA, L-Gln, PS, and 2-ME], or hepatic differentiation medium [DMEM supplemented with 10 ng/ml HGF (PEPROTECH), 1 μM DEX (SIGMA), 10% KSR, glucose 2000 mg/ml, NEAA, L-Gln, PS, and 2-ME], or pancreatic differentiation medium [RPMI supplemented with 0.25 μM KAAD-Cyclopamine (Stemgent), 50 ng/ml recombinant human FGF10 (PeproTech), 2% B27, NEAA, L-Gln, PS, and 2-ME for 2days, DMEM supplemented with 2 μM all-trans RA (Stemgent), 0.25 μM KAAD-Cyclopamine (Stemgent), 50 ng/ml recombinant human FGF10 (PeproTech), 10 μM SB431542 (Stemgent), 2% B27, NEAA, L-Gln, PS, and 2-ME for 6 days], or anterior foregut differentiation medium [DMEM supplemented with 10 μM SB431542, 1.5 μM Dorsomorphine (SIGMA), 2% B27, NEAA, L-Gln, PS, and 2-ME]. The medium was replaced every 2 days with fresh medium supplemented with growth factors.

### Antibodies

For immunocytochemical analysis, goat anti-SOX17 antibody (1:200, R&D systems, AF1924), rabbit anti-Hnf3b/Foxa2 (1:300, Millipore, 07–633), mouse anti-Cdx2 (1:500, BioGenex, MUC392-UC), rabbit anti-Cdx2 (1:4000, Abcam, ab765451), mouse anti-Villin (1:500, BD Transduction Laboratories, 610359), rabbit anti-GPR49/ Lgr5 (1:200, MBL, LS-A-1235) and mouse anti-SLC15A1/Pept1 (1:200, Abcam, ab58165) were used.

For flow cytometric analysis, PE/Cy7 anti-CD117 (1:50, BioLegend, 313212), APC anti-CXCR4 (1:25, BioLegend, 306510), mouse anti-Villin (1:200, BD Transduction Laboratories, 610359) rabbit anti-GPR49/ Lgr5 (1:200, MBL, LS-A-1235) and rabbit anti-Phospho-Smad2 (1:200, Cell signalling, 3101) were used.

For western blotting analysis, mouse anti-alpha-Tubulin (1:2000, Developmental Studies Hybridoma Bank, University of Iowa, 12G10), rabbit anti-Smad2 (1:1000, Cell signalling, 5399S), rabbit anti-Phospho-Smad2 (1:1000, Cell signalling, 3101) and mouse anti-Activated-beta-catenin (1:300, Millipore, 05–665) were used.

### Flow cytometry analysis

Cells were dissociated with Cell Dissociation Buffer (Invitrogen) and stained with the appropriate antibodies. The stained cells were recovered using FACSCanto (BD Pharmingen). Data were recorded using the BD FACS Diva Software program (BD Pharmingen) and analysed using the FlowJo program (Tree Star).

### Cell cycle analysis

Cells were dissociated with 0.25% trypsin. Dissociated cells were washed with PBS, then treated with Vybrant DyeCycle Ruby Stain (Life Technologies) for 30 min at 37 °C. Then, cells were analysed by FACSCanto.

### β-Ala-Lys(AMCA) intake

Cells were treated with 2.4 μM β-Ala-Lys(AMCA) at 37 °C for 4 hours.

## Additional Information

**How to cite this article**: Ogaki, S. *et al*. A cost-effective system for differentiation of intestinal epithelium from human induced pluripotent stem cells. *Sci. Rep*. **5**, 17297; doi: 10.1038/srep17297 (2015).

## Supplementary Material

Supplementary Information
