# Supplementary material for: A cost-effective system for differentiation of intestinal epithelium from human induced pluripotent stem cells
Source: Sci Rep. 2015 Nov 30;5:17297. doi: 10.1038/srep17297 (PMC4663490; doi:10.1038/srep17297)

A cost-effective system for differentiation of intestinal epithelium from human induced pluripotent stem cells

Soichiro Ogaki [1, 2, 3, 4, 5, *], Mayu Morooka[1, *], Kaito Otera[1] and Shoen Kume[§, 1, 3]

Supplementary Figure S1 DMSO enhances definitive endodermal differentiation from Toe.

Toe was used to confirm whether or not the DMSO potentiation of Activin-mediated definitive endoderm (DE) differentiation is conserved among cell lines. (A) A schematic representation of DE differentiation from Toe. (B, C) 0.8% DMSO promoted DE differentiation by Activin at 100 ng/ml, as shown by immunocytochemistry. Representative images are shown in C. Scale bar; 50 μm.

Supplementary Figure S2 DMSO enables a low concentration of Activin to induce DE differentiation from Toe.

Toe was used to confirm whether or not the DMSO potentiation of low dose Activin-mediated DE differentiation is conserved among cell lines. (A) A schematic representation of DE differentiation from Toe. (B) In the presence of 0.8% DMSO, Activin at 6.25 ng/ml could induce DE differentiation from Toe. Scale bar; 50 μm.

Supplementary Figure S3 DMSO was required during all periods of DE differentiation

The differentiation period was subdivided into 0–48 h and 48–96 h, during which periods 201B7 cells were differentiated by adding Activin alone (A) or in combination with DMSO (AD).

(A) DE differentiation was subdivided into 2 time windows, during which Activin (A) combined with/without DMSO (D) was added to the differentiation media. (B and C) Immunocytochemical analysis of SOX17 expression in cells treated with A and D during the 2 time windows are shown. Without DMSO Activin induced DE differentiation at low efficiencies from hiPS cells. Scale bar; 50 μm.

Supplementary Figure S4 DMSO promoted mesodermal differentiation.

We examined whether DMSO also promoted mesodermal differentiation from 201B7. (A) A schematic representation of mesodermal differentiation from 201B7. (B) 0.8% DMSO enhanced mesodermal differentiation induced by Activin at 100 ng/ml and BMP4 at 40 ng/ml. BRACHYURY-expressing cells increased by DMSO. (C and D) DMSO enabled a low concentration of BMP4 to induced mesoderm. (C) BMP4 at 2.5 ng/ml could not induced BRACHYURY-expressing cells. At the presence of 0.8% DMSO, Activin at 6.25 ng/ml and BMP4 at 2.5 ng/ml induced mesodermal differentiation of 201B7. (D) The representative immunofluorescence images of BRACHYURY-expressing cells. A6.25, Activn at 6.25 ng/ml. B2.5, BMP4 at 2.5 ng/ml. (E) Real-time PCR analysis of mesodermal markers. DMSO increased BRACHYURY and MESP1. Scale bar; 50 μm.

Supplementary Figure S5 Posterior endodermal cells from Toe

Immunocytochemistry of SOX17 and CDX2. DE induced by Activin in the presence of DMSO could differentiate into posterior endodermal cells. Scale bar; 100 μm.

Supplementary Figure S6 Other endodermal lineages were differentiated from hiPS cells by Activin at 6.25 ng/ml with DMSO

(A) DE induced by Activin at 6.25 ng/ml with DMSO was subjected to hepatic (Hep), pancreatic (Panc) or anterior foregut (AFG) differentiation. A low dose of Activin (6.25 ng/ml) with DMSO induced hiPS cells to differentiate into ALB^{+}/$CK19^{+}$ hepatoblasts (B), magnified images of the boxed areas depicted with white broken lines are shown (B). PDX1+ pancreatic progenitor (C) or SOX2+ anterior foregut cells (D). HepG2, a hepatocellular carcinoma cell line [S 1, S2], is used as a positive control of immunocytochemistry for ALB and CK19 expression. CYC; KAAD-Cyclopamine. RA; Retinoic acid. SB; SB431542. Nog; Noggin. Scale bar; 100 μm.

Supplementary Figure S7 Un-cropped gel images of western blotting and RT-PCR

Uncropped gel images of western blotting data in Figure 3 (A) and RT-PCR anlysis data in Figure 5 (B). (A) Lane 1; Control cells. Lane 2; Activin-treated cells. Lane3; DMSO-treated cells. Lane 4; Activin and DMSO-treated cells. The samples were derived from the same experiments and the gels were processed in parallel.(B) Lane 1; Day6. Lane 2; Day15. Lane 3; Fetal intestine (positive control). Lane 4; Adult intestine (positive control). Lane 5; DW, water (negative control). Lane 6; Activin-treated cells. Lane 7; Activin/DMSO-treated cells. Lane 8; DW (negative control). The samples (Day 6, 15, positive & negative controls) were derived from the same experiments and the gels were processed in parallel.

S1. Knowles BB, Howe CC, Aden DP. Human hepatocellular carcinoma cell lines secrete the major plasma proteins and hepatitis B surface antigen. *Science* **209**, 497-499 (1980).

S2. Yoneda N*, et al.* Epidermal growth factor induces cytokeratin 19 expression accompanied by increased growth abilities in human hepatocellular carcinoma. *Lab Invest* **91**, 262-272 (2011).

Table S1 Primer sequences used for RT-PCR analysis

Primer sequences used for detection of gene expression in Fig. 5.

Gene	Forward primer	Reverse primer
CDX2	GAGGGGGTGGTTATTGGACT	AGGAAGTCCAGGTTGGCTCT
VILLIN	ACTTCTATGGGGGCGACTG	ATGCGTCCCTTGAAGATGG
MDR1	CTTATGCTCTGGCCTTCTGG	GGAGATGCCTGTCCAACACT
MRP3	TCTGTCCTGGCTGGAGTCG	TCAGCTTGATGCGCGAGTC
OATP2B1	CTTCATCTCGGAGCCATACC	GCTTGAGCAGTTGCCATTG
EAAC1	CCTGGTGTCACCCAGAAAGT	GAAAACAGGCCTGGACAAGA
TAUT	AGGGAACTGAGGTGCAGAGA	CTGGAAGGAGAGCATCCAAG
CYP3A4	CAGGAGGAAATTGATGCAGTTTT	GTCAAGATACTCCATCTGTAGCACAGT
CYP2E1	GAAAACGAGTGTGTGCTGGA	CGGGGAATGACACAGAGTTT
CES2	AATCCCAGCTATTGGGAAGGA	CTGGCTGGTCGGTCTCAAAC
GAPDH	CGAGATCCCTCCAAAATCAA	CATGAGTCCTTCCACGATACCAA

Supplementary Figure. S1

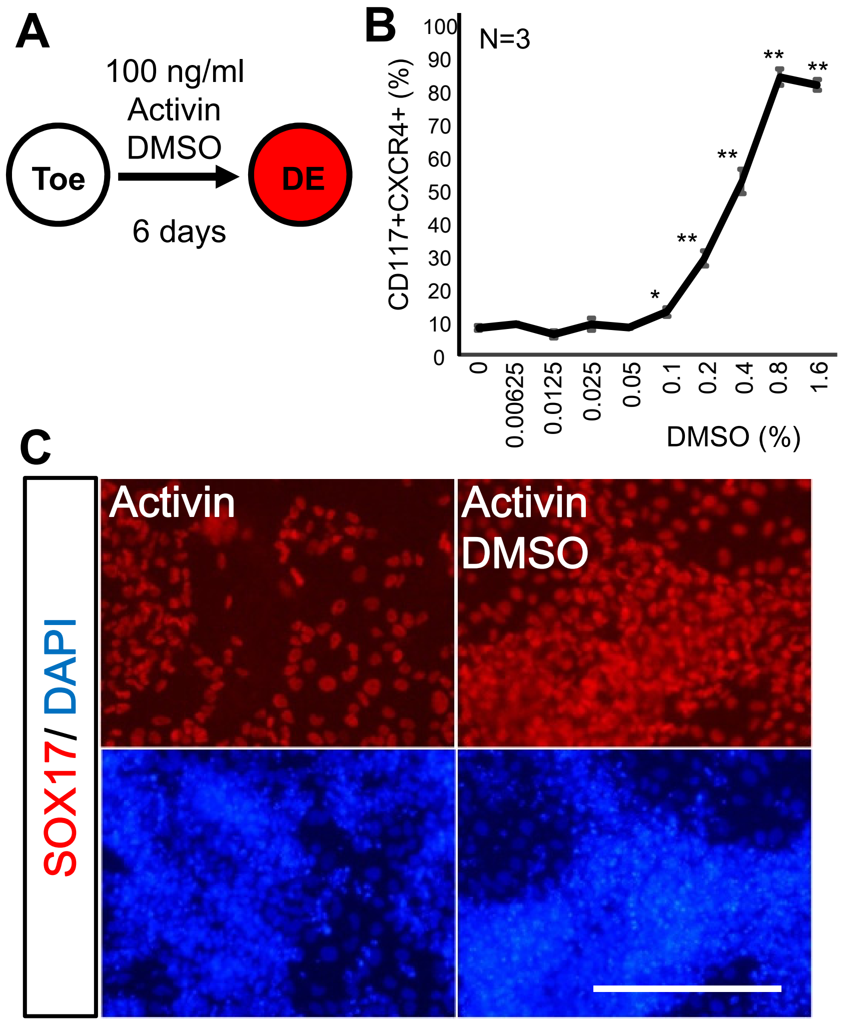

Supplementary Figure. S2

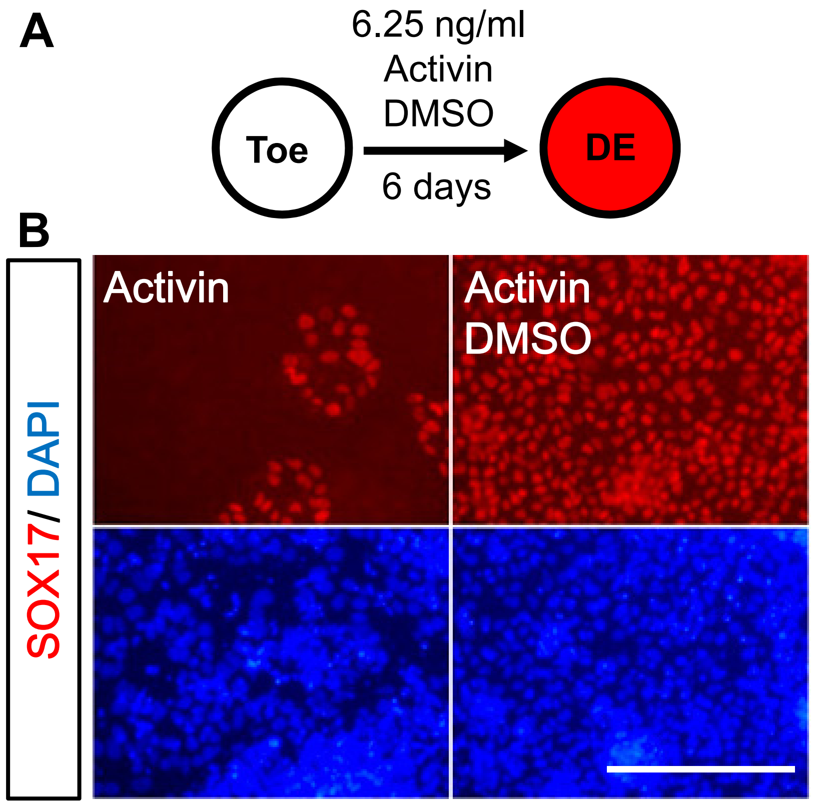

Supplementary Figure. S3

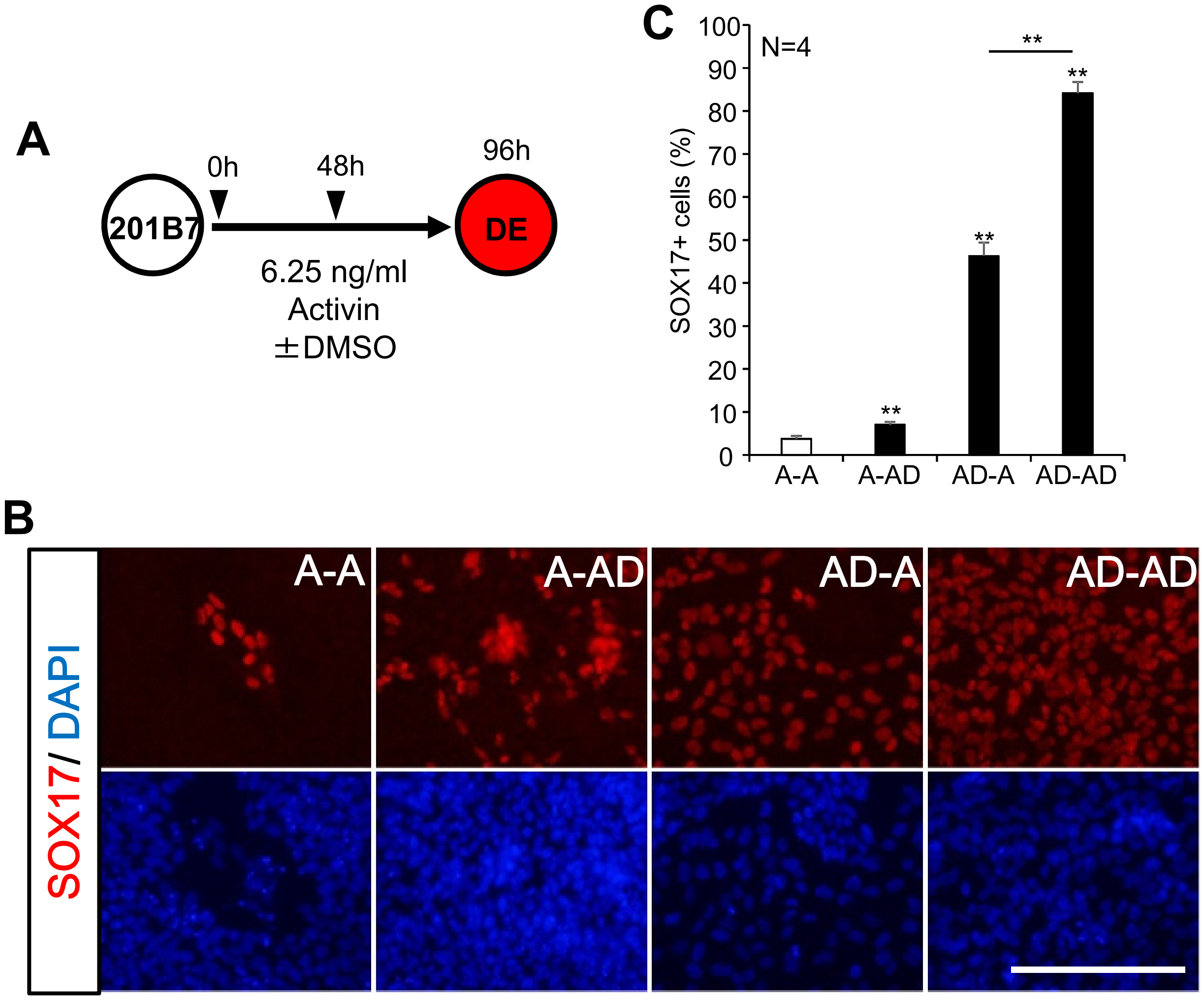

Supplementary Figure. S4

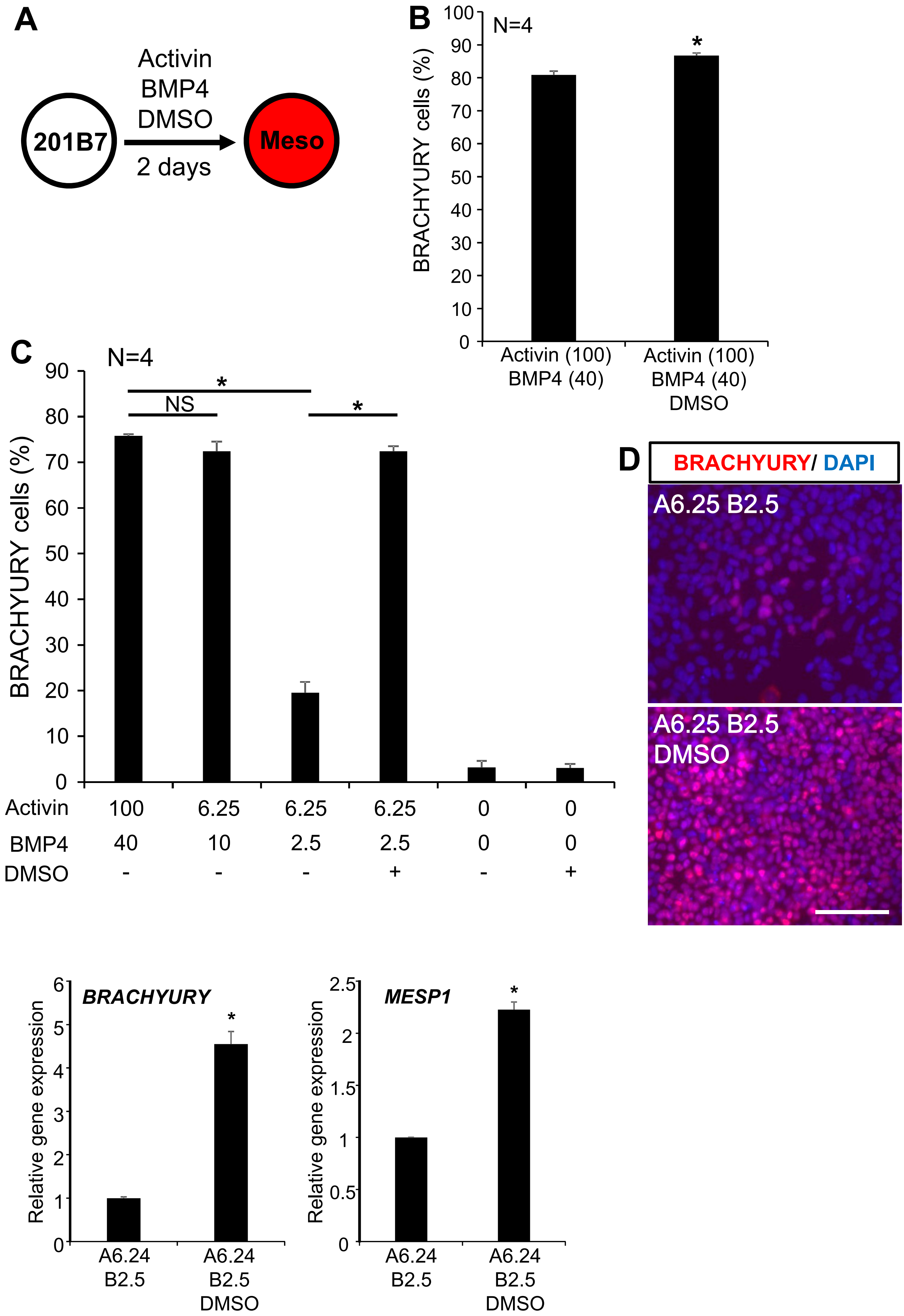

Supplementary Figure. S5

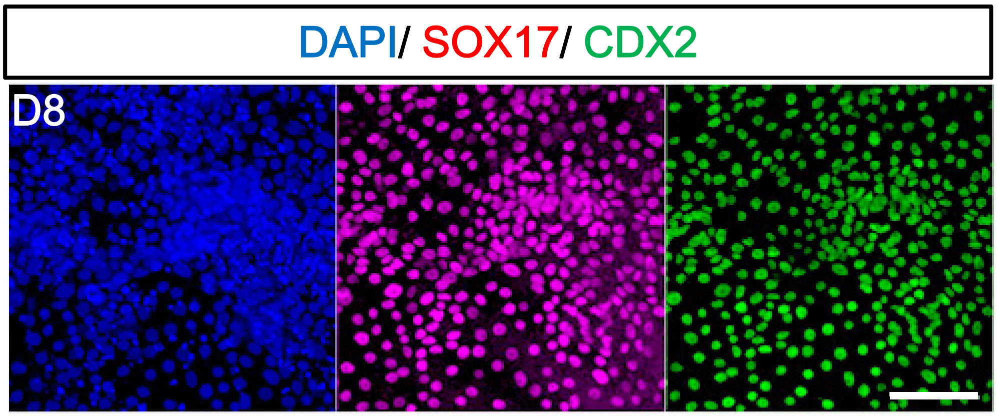

Supplementary Figure. S6

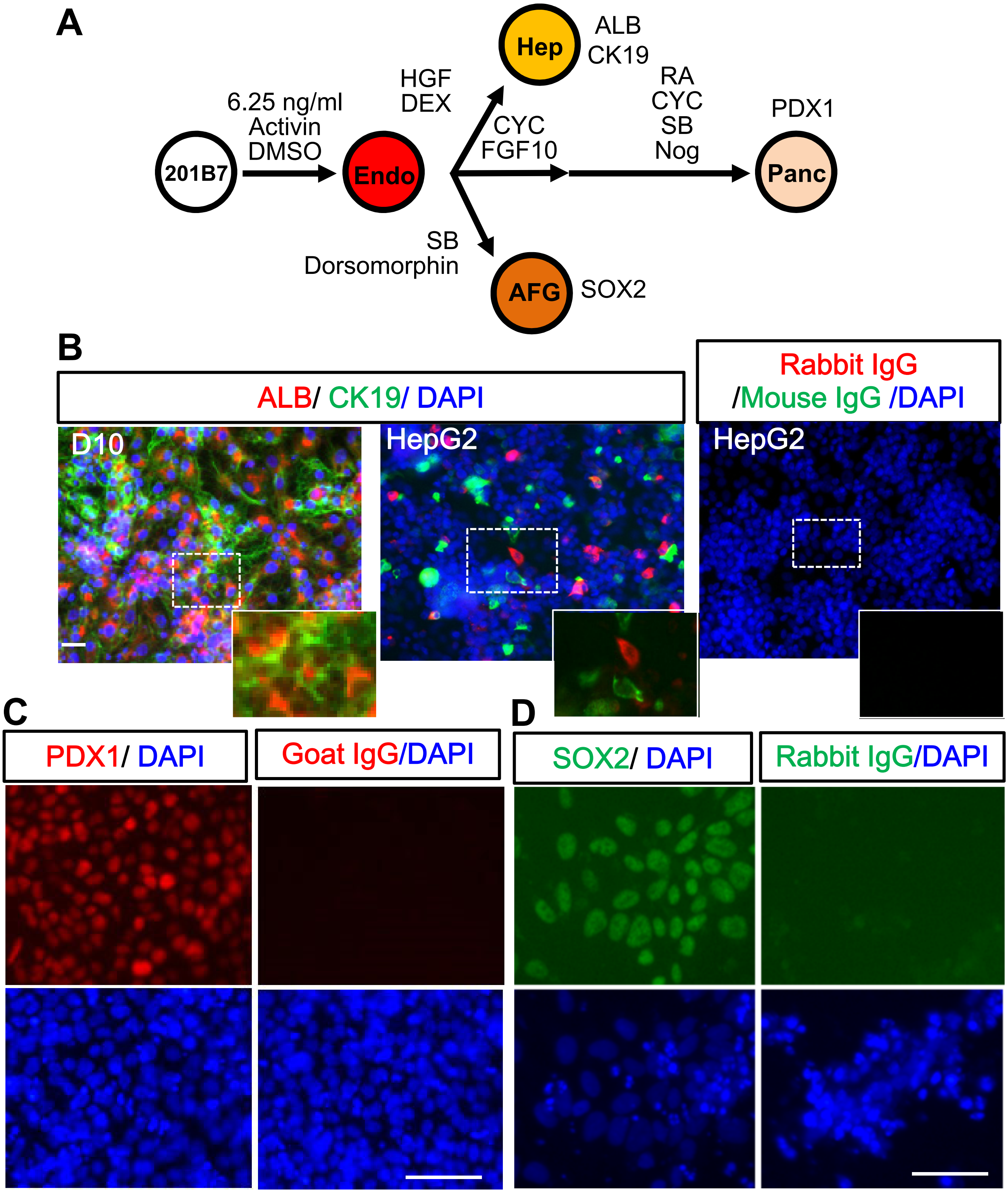

Supplementary Figure. S7

(A) Figure. 3

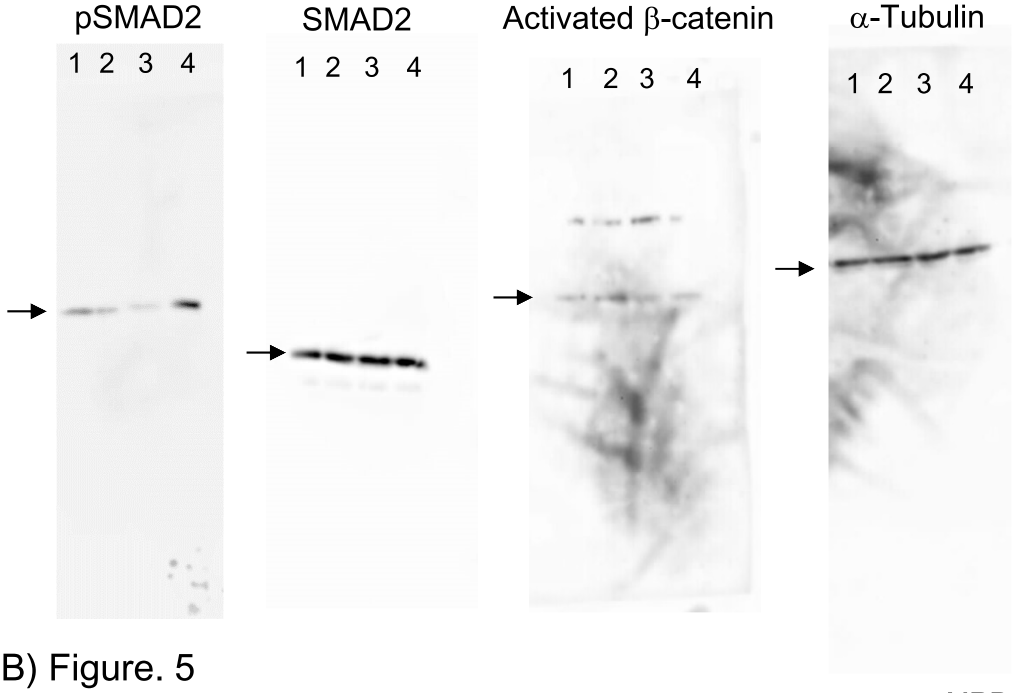

(B) Figure. 5

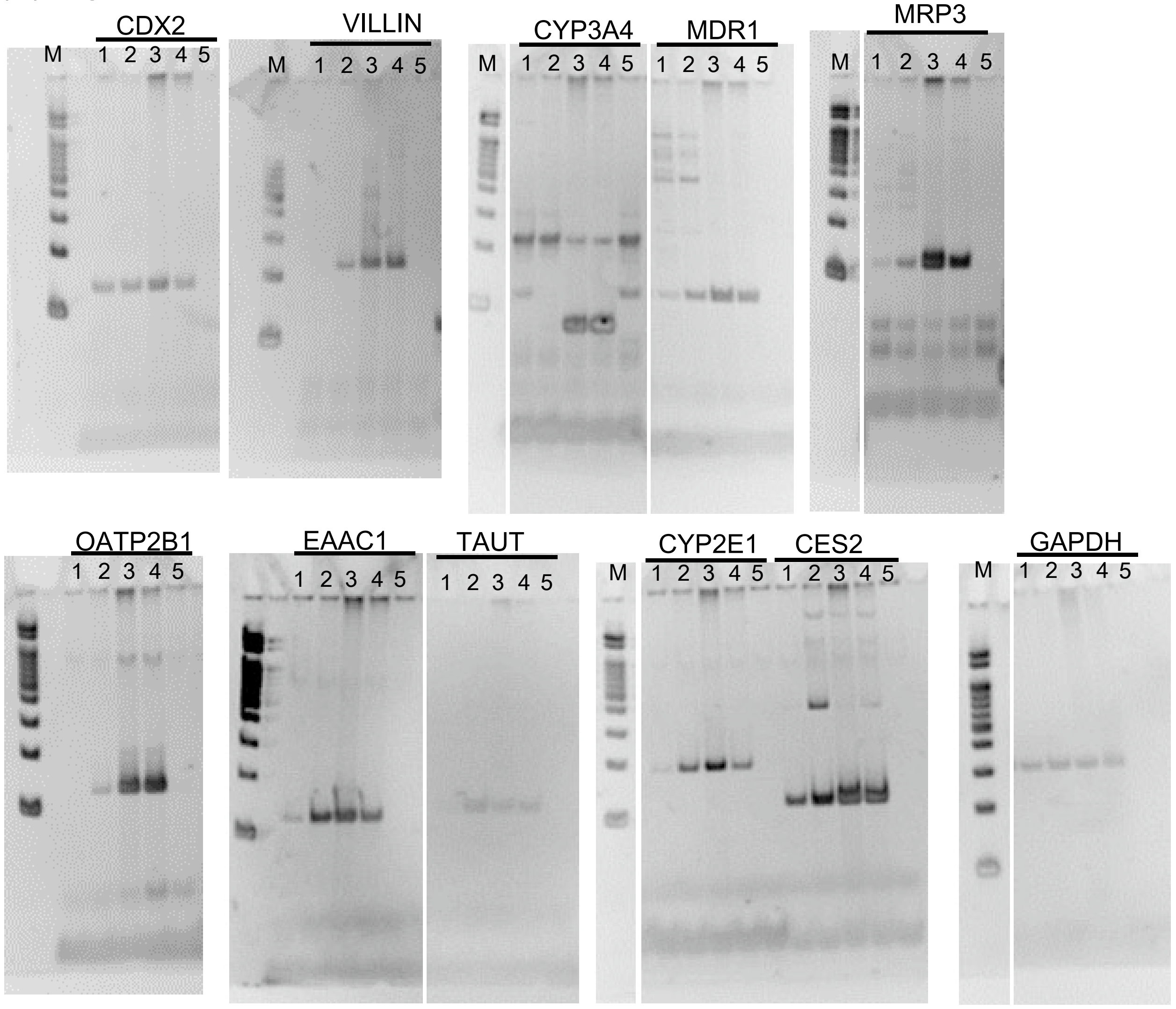

Supplement: Supplementary Information [file srep17297-s1.pdf]
